# Long Non-coding RNA TUG1 Sponges Mir-145a-5p to Regulate Microglial Polarization After Oxygen-Glucose Deprivation

**DOI:** 10.3389/fnmol.2019.00215

**Published:** 2019-09-10

**Authors:** Haoyue Wang, Songjie Liao, Hongjie Li, Yicong Chen, Jian Yu

**Affiliations:** Department of Neurology, National Key Clinical Department and Key Discipline of Neurology, Guangdong Provincial Engineering Center for Major Neurological Disease Treatment, Guangdong Provincial Translational Medicine Innovation Platform for Diagnosis and Treatment of Major Neurological Disease, First Affiliated Hospital of Sun Yat-sen University, Guangzhou, China

**Keywords:** taurine up-regulated gene 1, microRNA-145a-5p, inflammatory cytokines, NF-κB signaling, microglia, phenotype, oxygen-glucose deprivation

## Abstract

Microglia plays a critical role in neuroinflammation after ischemic stroke by releasing diverse inflammatory cytokines. Long non-coding RNA taurine up-regulated gene 1 (lncRNA TUG1) is widely expressed in adult brain and has been reported to participate in multiple biological processes associated with nervous system diseases. However, the role of TUG1 in microglial activation remains unidentified. BV-2 microglial cells were cultured *in vitro* and TUG1 siRNA was used to knock down its RNA level. Microglial cells were subjected to oxygen-glucose deprivation (OGD) for 4 h following TUG1 siRNA or scramble siRNA transient transfection. After 24 h reoxygenation, TUG1 level and microglial M1/M2 phenotype, as well as releasing inflammatory cytokines and their role to viability of SH-SY5Y neuroblastoma cells were determined by quantitative real-time PCR (qRT-PCR), ELISA, immunofluorescence and western blot. In addition, miR-145a-5p, a putative microRNA to bind with TUG1 by bioinformatics analysis, was simultaneously examined, then the interaction of TUG1 with miR-145a-5p and the potential involvement of NF-κB pathway were further evaluated by RNA-RNA pull-down assay and western blot. The cellular level of TUG1 was transiently up-regulated in microglial cells 24 h after OGD treatment, with an inverse correlation to downregulated miR-145a-5p. TUG1 knockdown drove microglial M1-like to M2-like phenotypic transformation with reduced production of pro-inflammatory cytokines (tumor necrosis factor-α, TNF-α; interleukin-6, IL-6) and incremental release of anti-inflammatory cytokine (interleukin-10, IL-10), as a result, promoted the survival of SH-SY5Y cells. Meanwhile, TUG1 knockdown prevented OGD-induced activation of NF-κB pathway as well, represented by decreased ratios of p-p65/p65 and p-IκBα/IκBα proteins. Furthermore, we found that TUG1 could physically bind to miR-145a-5p while miR-145a-5p inhibitor abolished the protective effects of TUG1 knockdown through activation of NF-κB pathway, suggesting a negative interaction between TUG1 and miR-145a-5p. Our study demonstrated that lncRNA TUG1, sponging miR-145a-5p with negative interaction, could regulate microglial polarization and production of inflammatory cytokines at a relatively early stage after OGD insult, where NF-κB pathway might be involved, possibly providing a promising therapeutic target against inflammatory injury.

## Introduction

Ischemic stroke is one of the leading causes of death and disability worldwide, with limited treatment options available (Powers et al., 2018). Innate and adaptive neuroinflammation in the brain, characterized by microglial activation, production of cytokines and infiltration of peripheral immune cells, occurs over a time course spanning from minutes to weeks after stroke (Anrather and Iadecola, 2016). The post-ischemic inflammation is conventionally considered detrimental to brain parenchyma and previous studies of neuroprotective therapies mainly focused on preventing the functions of immune cells and inflammatory mediators. However, increasing evidence indicates that post-ischemic inflammation could be also beneficial by alleviating neuronal apoptosis and strengthening neurogenesis, exclusive suppression of which may compromise the neuroprotective effects (Jin et al., 2013; Fu et al., 2015). Therefore, immunomodulation to promote beneficial effects, instead of simple immunosuppression, would be a more reasonable therapy strategy for stroke.

Long non-coding RNAs (lncRNAs), a group of untranslated regulatory RNA molecules with more than 200 bases, have been demonstrated to modulate almost all the protein-coding genes through chromatin modification, transcriptional or post-transcriptional processing (Mercer and Mattick, 2013; Chen and Zhou, 2017). A large number of lncRNAs are highly expressed in central nervous system with discrepant distribution in neuroanatomical regions and, although lack of protein-coding function, considered as important regulators in vascular and neural damage in multiple neurological diseases including stroke (Qureshi and Mehler, 2012; Bhattarai et al., 2017; Bao et al., 2018). In particular, lncRNA taurine up-regulated gene 1 (TUG1), a taurine-induced lncRNA essential to the forming of photoreceptors in retinal development (Young et al., 2005), was up-regulated in brain ischemic penumbra and sponged microRNA-9 to promote neuronal apoptosis (Chen et al., 2017). However, the role of TUG1 to neuroinflammation remains to be unraveled. On the other hand, microRNAs (miRNAs), another class of non-coding single-stranded RNAs with 18–24 nucleotides, can serve as a gene silencer or a translation inhibitor by binding to the target mRNAs (Bhalala et al., 2013). For example, miR-145, a tumor suppressor expressed in various tumors, has recently been demonstrated to participate in cell apoptosis and inflammatory response (Yuan et al., 2017; Ye et al., 2019). Of note, lncRNAs was shown to be able to directly bind to specific miRNAs to regulate post-transcriptional processing through competing endogenous RNA mechanism (Wilusz et al., 2009). Thereby, we also focus on the interaction between TUG1 and miRNAs.

In the present study, bioinformatics analysis discovered putative binding sites of TUG1 with miR-145a-5p, implying TUG1 might function as a sponge for miR-145a-5p. Subsequently, we detected the levels of TUG1 and miR-145a-5p in microglial cells after oxygen-glucose deprivation (OGD) and ascertained their binding relationship by RNA-RNA pull-down assay. Furthermore, we explored the functional involvement of TUG1 in microglial polarization and their regulation to NF-κB pathway, a well-known inflammatory mediator.

## Materials and Methods

### Cell Culture and OGD

BV-2 microglial cells (Kunming Cell Bank, Chinese Academy of Sciences) and SH-SY5Y human neuroblastoma cells (ATCC, Manassas, VA, USA) were respectively grown in Dulbecco’s modified eagle medium supplemented with 10% fetal bovine serum and 1% penicillin/streptomycin in a humidified incubator under 5% CO_2_ at 37°C. Cells were split at 70%–80% confluence before the following experiments.

OGD was induced to mimic ischemic conditions *in vitro* as previously described (Yu et al., 2017). Briefly, BV-2 microglial cells were maintained at 37°C with glucose-free Dulbecco’s modified eagle medium in a modular chamber with dual flow meter (Billups-Rothenberg, Del Mar, CA, USA), and then flushed with 95% N_2_/5% CO_2_ gas mixture at a flow rate of 4L/min for 10 min to create hypoxic condition. Hypoxic condition within the chamber was monitored using a gas analyzer (Coy Laboratory, Grass Lake, MI, USA). Thereafter, cells were transferred to normal culture medium for an additional 12, 24 or 48 h under 5% CO_2_ at 37°C for reoxygenation. The extent of OGD-induced death of cells was dependent on the duration of OGD and reoxygenation, and OGD for 4 h and reoxygenation for 24 h was at a critical threshold to induce pivotal signaling events for cells without causing excessive cell death in the current regimen. Control cells were treated without OGD condition.

### Cell Transfection

TUG1 small interfering RNA (siRNA) and miR-145a-5p inhibitor, as well as their corresponding negative controls (NCs) were designed by GenePharma Corporation (Suzhou, China). BV-2 microglial cells (1.5 × 10^5^/well) in a 6-well plate were transfected with 200 pmol TUG1 siRNA, 100 pmol miR-145a-5p inhibitor or their NCs by using a Lipofectamine 2,000 reagent (Invitrogen, Carlsbad, CA, USA) according to the manufacturer’s instructions. Transfected cells were incubated for an additional 24 h prior to OGD treatment. Corresponding Sequences were as follows: TUG1 siRNA-sense, 5′-CCAUCUCACAAGGCUUCAATT-3′, antisense, 3′-TTGGUAGAGUGUUCCGAAGUU-5′; si NC-sense, 5′-UUCUCCGAACGUGUCACGUTT-3′, antisense, 3′-ACGUGACACGUUCGGAGAATT-5′; miR-145a-5p inhibitor, 5′-AGGGAUUCCUGGGAAAACUGGAC-3′; mi NC, 5′-CAGUACUUUUGUGUAGUACAA-3′. The efficiency of transfection was validated by comparing the levels of TUG1 and miR-145a-5p between transfected and controlled cells by quantitative real-time-polymerase chain reaction (qRT-PCR).

### qRT-PCR

Total RNA from BV-2 microglial cells was extracted using a RNAzol RT reagent (MRC, OH, USA), then the cDNA was synthesized using a PrimeScript™ RT reagent Kit (Takara, China) according to the manufacturer’s protocols. The relative mRNA levels of TUG1 and miR-145a-5p were measured in 2^−ΔΔCT^ method using a TB Green™ Premix Ex Tag™ II kit (Takara) on ABI 7,500 real-time system (Applied Biosystems, Foster, CA, USA). The 2^−ΔΔCT^ method to normalize gene expression data was achieved as described previously (Livak and Schmittgen, 2001). GAPDH was chosen as an internal reference gene for TUG1 while U6 for miR-145a-5p. The target gene levels showed by CT values were calculated relative to the internal reference genes. All samples were performed at least three parallel reactions. Primer sequences were as follows: TUG1-forward: 5′-TGCCCAATTCCACCAAGGAA-3′, reverse: 5′-CTGCCAACCTTCTATACGCCT-3′; GAPDH-forward: 5′-TGTGTCCGTCGTGGATCTGA-3′, reverse: 5′-TTGCTGTTGAAGTCGCAGGAG-3′. The primers for U6 and miR-145a-5p were designed by Ribobio Corporation (Guangzhou, China) and quantified by a Bulge-Loop™ miRNAs qPCR Primer kit (Ribobio). The design of primer sequences by Ribobio has acquired a Chinese patent (licensed No. ZL2014-1-0039162.6), which is not publicly available. However, it has been widely applied in a large number of published articles (Liu et al., 2012; Xia et al., 2018; Chen et al., 2019).

### Immunofluorescence

To detect microglial phenotypes, BV-2 microglial cells (2.5 × 10^4^/well) in a 24-well plate were first dispensed on a 12 mm-diameter coverslip to assure about 70% cell density. A mouse anti-mouse ARG1 antibody (1:500, Santa Cruz Biotechnology, Santa Cruz, CA, USA) or rat anti-mouse CD68 (1:1,000, Abcam, Cambridge, MA, USA) was used to mark M1-like microglial phenotype. A rabbit anti-mouse CD206 or rat anti-mouse CD16 (1:1,000, Abcam) was used to mark M1-like microglial phenotype. Species-specific Alexa Fluor 488 or 555-conjugated anti-rat, anti-mouse, or anti-rabbit secondary antibodies (1:500, CST, Danvers, MA, USA) was used to detect any positive signal, respectively. Thereafter, cells were counterstained with DAPI. The reaction product was absent when the primary antibody was omitted. The number of microglial cells labeled with positive M1-like or M2-like signal as well as the total number of cells was respectively counted in five randomly selected fields under 40× magnification by using a laser confocal microscope (Nikon DS-Ri2, Japan). Then, the percentage of positive cell number relative to the total cells was calculated in different treatment groups.

### Western Blot

BV-2 microglial cells (1.5 × 10^5^/well) in a 6-well plate were lysed using RIPA buffer plus 10 μl/mL protease inhibitor cocktail (Thermo Fisher Scientific, Waltham, MA, USA) to extract total proteins. The protein concentration of each sample was quantified using a BCA protein assay kit (Thermo Fisher Scientific, Waltham, MA, USA). Equivalent amount of proteins was resolved by SDS-PAGE and transferred to polyvinylidene fluoride membranes. Blots were incubated at 4°C overnight with primary antibodies specific to ARG1 (1:500, Santa Cruz Biotechnology); CD68, CD206, CD16 (1:1,000, Abcam); rabbit anti-mouse p65, p-p65, p-IκBα, β-tubulin, β-actin, or mouse anti-mouse IκBα (1:1,000, CST). Antibodies binding to blots were visualized on X-ray films using a HRP-linked secondary antibody kit. Because the molecular weight of CD16 and β-tubulin is around 55 KD and that of β-actin, IκBα and p-IκBα is around 40 KD, β-actin was chosen as an internal reference for CD16 while β-tubulin for IκBα and p-IκBα. The image program of Quantity One was used to measure the density of bands in a blinded manner. Results were expressed as a percentage of β-tubulin or β-actin to generate relative protein levels.

### Detection of Cytokine Level and Cell Viability

The cultured supernatant of BV-2 microglial cells (1.5 × 10^5^/well) in a 6-well plate in different treatment groups was collected. The concentrations of tumor necrosis factor-α (TNF-α), interleukin-6 (IL-6) and interleukin-10 (IL-10) in the supernatant were quantified using a commercial ELISA kit (Novus Biologicals, Centennial, CO, USA) according to the manufacturer’s instructions.

To evaluate the role of cytokines to cell viability, SH-SY5Y cells (10^4^/well) were dispensed in a 96-well plate and pre-incubated for 24 h at 37°C, then cells in each well were mixed with 100 μl cultured supernatant of BV-2 microglial cells in different treatment groups and incubated for 2 h at 37°C. Viability of SH-SY5Y cells was detected by using a cell counting kit-8 (CCK-8, Dojindo, Tokyo, Japan) following the instructions of manufacturer. Blank control in non-cell well was simultaneously performed.

### RNA-RNA Pull-Down Assay

RNA-RNA pull-down was used to detect potential binding between TUG1 and miR-145a-5p. In brief, DNA fragment for TUG1 and corresponding NC were PCR amplified using T7-containing promoter and then cloned into Fast-T1 competent cells (Sagene, Guangzhou, China). RNA sequence produced from the amplified region of TUG1 was biotinylated using TranscriptAid T7 High Yield Transcription Kit and Bio-16-UTP (Thermo Fisher Scientific, Waltham, MA, USA) and then mixed with the lysate of BV-2 cells for 2 h at 4°C to form TUG1-RNA complexes. Thereafter, the complexes were incubated with 50 μl pre-washed Dynabeads Myone Streptavidin C1 (Thermo Fisher Scientific, Waltham, MA, USA) for 2 h to capture biotin-labeled TUG1 RNA. Finally, the complexes of RNA bound to beads were eluted and the level of TUG1-bound miR-145a-5p was examined with qRT-PCR. To further verify the binding between TUG1 and miR-145a-5p, a reverse RNA-RNA pull-down was also performed and the level of miR-145a-5p-bound TUG1 was examined with qRT-PCR as described above.

### Statistical Analyses

All data were analyzed with SPSS software (SPSS Inc., Chicago, IL, USA). All analysis was performed in a blinded manner without knowledge of the treatment assignment. Numerical data were expressed as the mean ± standard deviation. Student’s *t*-test or a general linear model with Bonferroni correction for analysis of variance was employed in the comparison of individual measurements when appropriate. A two-tailed *P*-value of 0.05 or less was taken to infer statistical significance.

## Results

### OGD Resulted in Transient Up-Regulation of TUG1 and Down-Regulation of Mir-145a-5p

**Figure 1:**
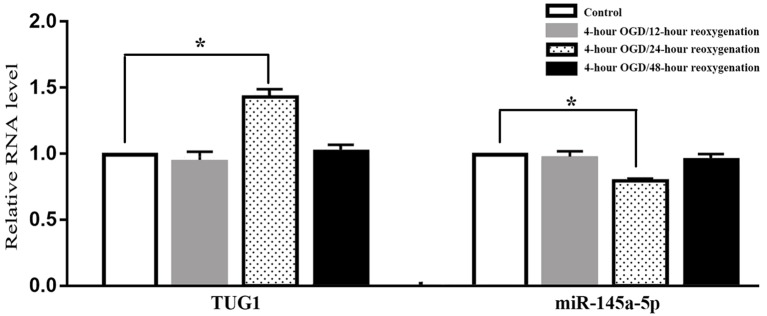
Quantification of RNAs by quantitative real-time-polymerase chain reaction (qRT-PCR), displaying transiently up-regulated taurine up-regulated gene 1 (TUG 1) concomitant with down-regulated miR-145a-5p after 4-h OGD and 24-h reoxygenation. No significant difference was observed at 12-h or 48-h reoxygenation. *P < 0.05 vs. the corresponding control. OGD, oxygen-glucose deprivation. *n* = 6.

When compared with normal control, the level of TUG1 detected by qRT-PCR was transiently up-regulated to 1.4-fold after 4-h OGD and 24-h reoxygenation. Meanwhile, the level of miR-145a-5p was transiently down-regulated to 65 percent of the normal level, implying an inverse correlation between TUG1 and miR-145a-5p. There was no significant difference between the groups with 12-h or 48-h reoxygenation after OGD and normal control (Figure 1, *n* = 6). Therefore, cells reoxygenated for 24 h were used in further experiments.

### TUG1 Acted as a Molecular Sponge for Mir-145a-5p

**Figure 2:**
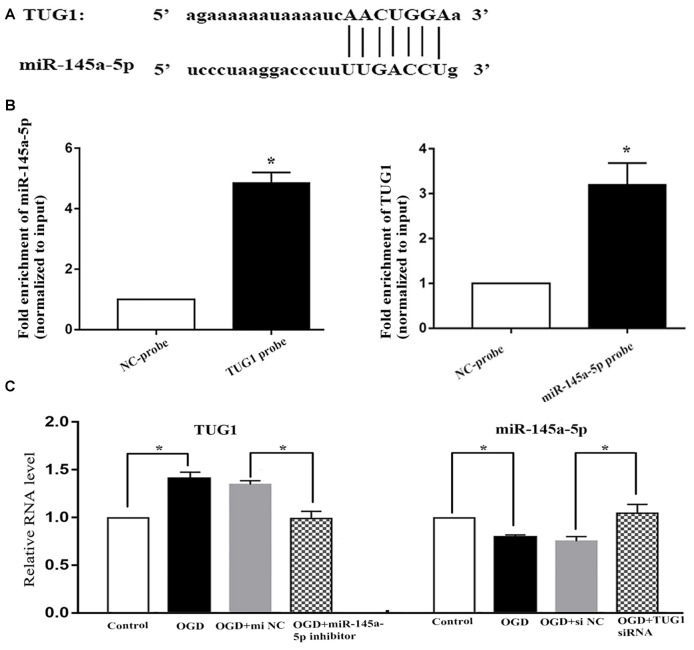
Correlation between TUG1 and miR-145a-5p. **(A)** Putative binding sites revealed by bioinformatics analysis. **(B)** Direct binding further confirmed by biotin-labeled RNA-RNA pull-down assay. **(C)** Quantification of RNAs by qRT-PCR. OGD-induced up-regulation of TUG1 was sufficiently turned over by miR-145a-5p inhibitor, vice versa, OGD-induced down-regulation of miR-145a-5p was sufficiently turned over by TUG1 siRNA, suggesting a negative interaction between TUG1 and miR-145a-5p. *P < 0.05 vs. the corresponding control. NC, negative control. *n* = 4.

Online bioinformatics analysis (starbase v3.0) discovered putative binding sties of TUG1 with miR-145a-5p (Figure 2A). The direct binding was further affirmed by biotin-labeled RNA-RNA pull-down system, where 4.9-fold increased amount of miR-145a-5p in TUG1 pulled down pellet and 3.2-fold increased amount of TUG1 in miR-145a-5p pulled down pellet were observed by qRT-PCR compared with control group (Figure 2B, *n* = 4). Meanwhile, OGD-induced up-regulation of TUG1 was sufficiently turned over by miR-145a-5p inhibitor, vice versa, OGD-induced down-regulation of miR-145a-5p was adequately turned over by TUG1 siRNA, suggesting that TUG1 could specifically sponge miR-145a-5p with negative interaction (Figure 2C, *n* = 4).

### TUG1 Knockdown Drove Microglial M1-Like to M2-Like Polarization After OGD, Reversed by Mir-145a-5p Inhibitor

**Figure 3:**
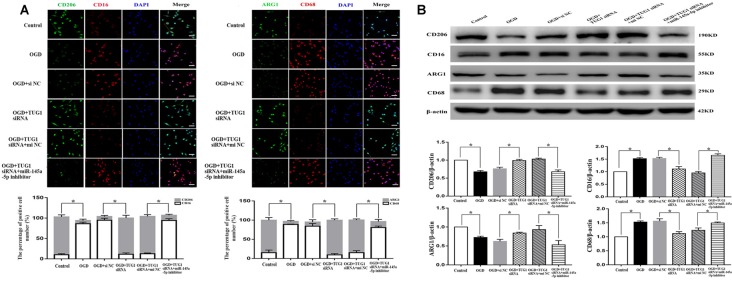
Microglial polarization by immunofluorescence **(A)** and western blot **(B)**. TUG1 knockdown increased the number of ARG1 and CD206 positive cells (M2-like phenotype, green) as well as the protein levels, whereas decreased that of CD16 and CD68 positive cells (M1-like phenotype, red) as well as the protein levels after OGD. The effect of TUG1 knockdown on microglial polarization was reversed by miR-145a-5p inhibitor. *P < 0.05 vs. the corresponding control. 400×. Scale bar = 50 μm. *n* = 4.

The effect of TUG1 to microglial polarization was determined by immunofluorescence and western blot. OGD increased the number of CD 16 and CD68 (M1-like phenotype markers) positive cells, with ~1.5-fold up-regulation of CD16 and CD68 protein levels, and decreased the number of CD206 and ARG1 (M2-like phenotype markers) positive cells, with 30 percent down-regulation of ARG1 and CD206 protein levels. By contrast, TUG1 knockdown significantly diminished the percentage of M1-like microglia from ~94% to ~11% and augmented that of M2-like microglia from ~9% to ~89% after OGD (Figure 3A, *n* = 4). Similarly, OGD-induced up-regulation of CD16 and CD68 proteins and down-regulation of ARG1 and CD206 proteins were efficiently reversed by TUG1 knockdown as well (Figure 3B, *n* = 4). After co-transfection with miR-145a-5p inhibitor, the effect of TUG1 knockdown on microglial polarization was reversed compared with inhibitor control, indicating that TUG1/miR-145a-5p regulated microglial polarization.

### TUG1 Knockdown Prevented OGD-Induced Inflammatory Injury to SH-SY5Y Cells, Reversed by Mir-145a-5p Inhibitor

**Figure 4:**
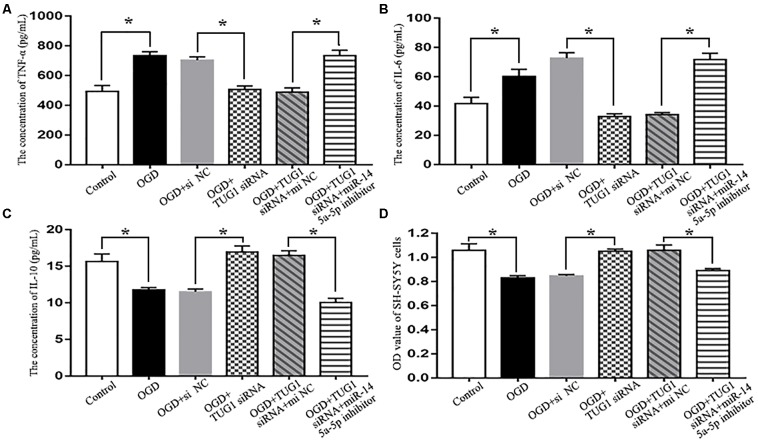
Cytokine detection by ELISA and cell viability assay by CCK-8. TUG1 knockdown suppressed the production of tumor necrosis factor-α (TNF-α; **A**) and interleukin-6 (IL-6; **B**) while elevated the concentration of interleukin-10 (IL-10; **C**) after OGD, with enhanced SH-SY5Y cell viability after OGD **(D)**. The effects of TUG1 knockdown on inflammatory cytokines and cell survival were abolished by miR-145a-5p inhibitor. *P < 0.05 vs. the corresponding control. *n* = 4.

When compared with normal control, OGD increased the concentrations of pro-inflammatory cytokines TNF-α and IL-6 (734.0 ± 52.8 vs. 493.9 ± 79.2 pg/mL, 60.2 ± 8.5 vs. 41.6 ± 7.5 pg/mL, respectively), and decreased that of anti-inflammatory cytokine IL-10 (11.8 ± 0.5 vs. 15.7 ± 1.8 pg/mL) in the cultured supernatant of BV-2 microglial cells, which was detrimental to the survival of SH-SY5Y cells (0.83 ± 0.03 vs. 1.06 ± 0.08 by OD value). By contrast, TUG1 knockdown significantly suppressed OGD-induced production of TNF-α and IL-6 (507.1 ± 47.9 pg/mL, 32.8 ± 3.2 pg/mL, respectively), elevated the concentration of IL-10 (16.9 ± 1.4 pg/mL), thus, promoted the survival of SH-SY5Y cells (1.05 ± 0.03 by OD value). After co-transfection with miR-145a-5p inhibitor, the beneficial effects of TUG1 knockdown on inflammatory cytokines and cell survival were significantly reversed compared with inhibitor control (Figure 4, *n* = 4).

### NF-κB Pathway Was Involved in TUG1/mir-145a-5p-Mediated Inflammatory Response

**Figure 5:**
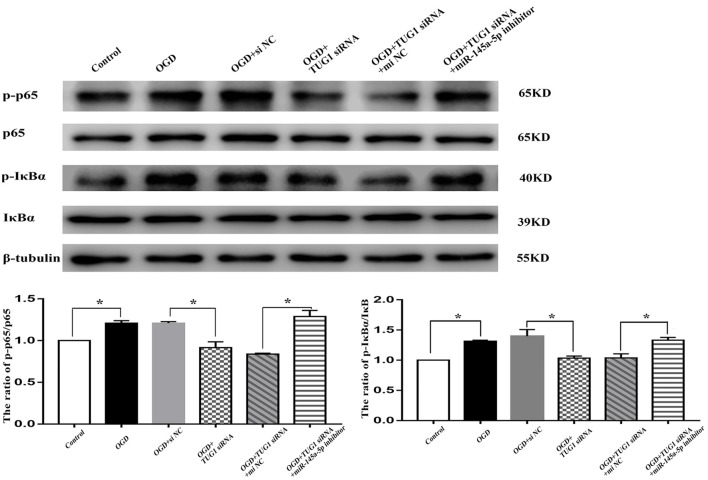
NF-κB activity by western blot analysis. OGD-induced NF-κB activation was inhibited by TUG1 knockdown, represented by the decreased ratios of p-p65/p65 and p-IκBα/IκBα proteins. The effect of TUG1 knockdown was reversed by miR-145a-5p inhibitor. *P < 0.05 vs. the corresponding control. *n* = 4.

The activity of NF-κB, represented by the ratios of p-p65/p65 and p-IκBα/IκBα, was enhanced to 1.2-fold and 1.3-fold, respectively after OGD. TUG1 knockdown significantly inhibited OGD-induced NF-κB activation compared with control siRNA. Meanwhile, miR-145a-5p inhibitor co-transfection distinctly abrogated the effect of TUG1 knockdown on NF-κB activation compared with inhibitor control (Figure 5, *n* = 4). The data implied an involvement of NF-κB pathway in TUG1/mir-145a-5p-mediated inflammatory response.

## Discussion

Resident microglia, the predominant immune cells in the brain, is rapidly mobilized to the lesion site, initiating the production of inflammatory mediators after ischemia. Microglial activation exhibits divergent effects in inflammatory response, where classic M1 phenotype releases detrimental pro-inflammatory mediators while alternative M2 phenotype releases protective anti-inflammatory mediators (Franco and Fernández-Suárez, 2015; Chen and Trapp, 2016; Ma Y. et al., 2017). Though alternative M2 polarization was beneficial to neurogenesis, transient M2 polarization at early stages after cerebral ischemia was gradually transformed into the M1 polarization in peri-infarct region (Hu et al., 2012; Wang et al., 2018). Thus, M2 polarization could be a promising target for stroke therapy.

LncRNAs participate in multiple biological processes by managing protein synthesis, RNA maturation and transport (Rinn and Chang, 2012; Kour and Rath, 2017). TUG1 has been regarded as an oncogenic lncRNA and aberrant up-regulation of TUG1 was closely related to different kinds of tumors (Li et al., 2016; Ma P. J. et al., 2017). In limited studies of non-neoplastic diseases, the function of TUG1 was still uncertain. TUG1 was up-regulated and shown to promote cell apoptosis in brain ischemia and atherosclerosis (Chen et al., 2016; Chen and Zhou, 2017). Nevertheless, TUG1 was also found downregulated and overexpression of TUG1 protected against cold-induced liver damage by inhibiting apoptosis (Su et al., 2016), indicative of its tissue-specific function to injury. By now, the role of TUG1 to microglial activation has not reported yet. In the present study, despite of no differences at 12 and 48 h after OGD, TUG1 was distinctly up-regulated 24 h after OGD, in consistent with increased M1-like phenotype markers CD16 and CD68. Moreover, pro-inflammatory cytokines TNF-α and IL-6, which was detrimental to the survival of SH-SY5Y cells, were also enhanced. It can be deduced that TUG1 was correlated with M1-like polarization. Indeed, we further observed that TUG1 knockdown diminished M1-like phenotype, whereas augmented M2-like phenotype markers ARG1 and CD206 as well as anti-inflammatory cytokine IL-10 and survival of SH-SY5Y cells. Taken together, the data indicated that TUG1 contributed to OGD-induced microglial M1-like polarization at a relatively early stage and inhibition of TUG1 was protective against inflammatory injury.

The underlying mechanism of TUG1 on microglial polarization is not clear. Accumulating evidence have revealed that lncRNAs can act not only as guide mediators and scaffolds for proteins to control the formation of cellular substructures but also as regulators of miRNAs to target mRNAs through sponge or bait ways (Ramos et al., 2016; Andersen and Lim, 2018). Previous studies indicated that miR-145 functioned as an inflammatory regulator in airway, vascular smooth muscle and retinal endothelial cells (Guo et al., 2016; O’Leary et al., 2016; Hui and Yin, 2018; Li et al., 2018). Moreover, overexpression of miR-145 was able to inhibit inflammatory injury after ischemia (Qi et al., 2017). It urged us to search a potential correlation between TUG1 and miR-145. After confirming a direct binding by RNA-RNA pull-down assay, we found an inverse correlation between TUG1 and miR-145a-5p after OGD and TUG1 knockdown. In addition, miR-145a-5p inhibitor abrogated the beneficial effects of TUG1 knockdown on microglial polarization, inflammatory cytokines and cell survival, showing that TUG1 could serve as a miR-145a-5p sponge with negative interaction.

MiR-145 has been proven to participate in various signaling transduction associated with multiple cellular processes such as cell proliferation, apoptosis and invasion by targeting downstream gene transcription (Cui et al., 2014; Vacante et al., 2019). Recent studies revealed that NF-κB pathway, a key mediator in inflammatory injury, was regulated by miR-145. Overexpression of miR-145 attenuated secretion of pro-inflammatory cytokines by suppressing NF-κB activity in high glucose-induced retinal endothelial cells (Hui and Yin, 2018), but accelerated pro-inflammatory cytokine reaction through activation of NF-κB signaling in atherosclerosis (Li et al., 2018), inferring diverse functions of miR-145 to NF-κB activity. In the present study, we found that TUG1 knockdown notably prevented OGD-induced activation of NF-κB pathway. With the administration of miR-145a-5p inhibitor, the effect of TUG1 knockdown was sufficiently abolished. Thus, the data implied that NF-κB pathway might be involved in TUG1/miR-145a-5p regulatory function.

There were several limitations in our study. First, we used BV-2 microglia instead of primary microglial cells for microglial study. Primary microglial cells are undoubtedly the best candidate for microglial study. However, BV-2 microglial cells have also been widely used for microglial study mostly due to the reservation of major characteristics and function of primary microglia (Kim et al., 2018, 2019; Kittl et al., 2018; Yang et al., 2018). Second, TUG1 regulated microglial activation by direct binding to miR-145a-5p with negative interaction, where NF-κB pathway might be involved, but the mechanisms how TUG1 decreased miR-145a-5p and how NF-κB pathway participated in the regulatory function of TUG1/miR-145a-5p were still unclarified. Future *in vivo* studies are warranted to explore these issues.

## Conclusion

In conclusion, the present study favors the results that lncRNA TUG1, sponging miR-145a-5p with negative interaction, could regulate microglial polarization and production of inflammatory cytokines at a relatively early stage after ischemic injury, where NF-κB signaling pathway might be involved, possibly providing a promising therapeutic target against inflammatory injury.

## Data Availability

The data used and/or analyzed during the current study will be available from the corresponding author on reasonable request.

## Author Contributions

HW and SL performed the experiment and analyzed the data. HL and YC analyzed the data. JY designed the work, drafted the manuscript, and interpreted the data. All authors read and approved the final manuscript.

## Conflict of Interest Statement

The authors declare that the research was conducted in the absence of any commercial or financial relationships that could be construed as a potential conflict of interest.

## Abbreviations

LncRNAs: Long non-coding RNAs
TUG1: Taurine up-regulated gene 1
miRNAs: MicroRNAs
OGD: Oxygen-glucose deprivation
siRNA: Small interfering RNA
NCs: Negative controls
TNF-α: Tumor necrosis factor-α
IL-6: Interleukin-6
IL-10: Interleukin-10.
